# A longitudinal study of queen health in honey bees reveals tissue specific response to seasonal changes and pathogen pressure

**DOI:** 10.1038/s41598-024-58883-1

**Published:** 2024-04-18

**Authors:** Duan C. Copeland, Vincent A. Ricigliano, Brendon M. Mott, Oliver L. Kortenkamp, Robert J. Erickson, June Gorrochategui-Ortega, Kirk E. Anderson

**Affiliations:** 1 USDA-ARS Carl Hayden Bee Research Center, 2000 E. Allen Rd, Tucson, AZ 85719 USA; 2 USDA-ARS Honey Bee Breeding, Genetics, and Physiology Laboratory, Baton Rouge, LA 70820 USA; 3 Department of Entomology and Center for Insect Science, University of Arizona, Tucson, AZ 85721 USA; 4 Department of Genetics, Physical Anthropology and Animal Physiology, University of the Basque Country (UPV/EHU), Barrio Sarriena S/N, 48940 Leioa, Spain

**Keywords:** Honey bee, Queen, Deformed wing virus, *Nosema*, Immune, *Melissococcus*, Microbiome, Pathogens, Infection, Microbial ecology

## Abstract

The health of honey bee queens is crucial for colony success, particularly during stressful periods like overwintering. To accompany a previous longitudinal study of colony and worker health, we explored niche-specific gut microbiota, host gene expression, and pathogen prevalence in honey bee queens overwintering in a warm southern climate. We found differential gene expression and bacterial abundance with respect to various pathogens throughout the season. Biologically older queens had larger microbiotas, particularly enriched in *Bombella* and *Bifidobacterium*. Both Deformed Wing Virus A and B subtypes were highest in the fat body tissue in January, correlating with colony *Varroa* levels, and Deformed Wing Virus titers in workers. High viral titers in queens were associated with decreased vitellogenin expression, suggesting a potential trade-off between immune function and reproductive capacity. Additionally, we found a complex and dynamic relationship between these viral loads and immune gene expression, indicating a possible breakdown in the coordinated immune response as the season progressed. Our study also revealed a potential link between *Nosema* and *Melissococcus plutonius* infections in queens, demonstrating that seasonal opportunism is not confined to just workers. Overall, our findings highlight the intricate interplay between pathogens, metabolic state, and immune response in honey bee queens. Combined with worker and colony-level metrics from the same colonies, our findings illustrate the social aspect of queen health and resilience over the winter dearth.

## Introduction

The honey bee (*Apis mellifera*) is a domesticated insect valued for its contributions to honey production and pollination services^1^. An observed trend of the last decade has amateur and commercial beekeeping operations in North America and Europe losing 30–50% of their hives annually^2^. These losses are attributed to a complex interplay of biotic and abiotic factors, often involving various pathogens and environmental stressors^3–5^. Among the leading causes of such losses are queen failures. Historically queens survived 2–5 years, but more recently, queen losses and supersedures occur much earlier and more frequently^6–10^. Now it’s common practice by many beekeepers to prophylactically replace queens on an annual basis, as younger queens are generally considered healthier and more productive^2,11,12^. The health and vitality of a honey bee colony depends on the well-being of its queen, who, as the sole egg-laying individual in the hive, plays a critical role in determining the colony's overall success. Amidst the myriad of challenges that honey bee colonies face, the overwintering period stands out as a time of heightened vulnerability. Overwintering is a particularly stressful period for honey bee colonies, marked by reduced foraging opportunities and increased susceptibility to pathogens. In the US, colonies that overwinter in warm southern climates like Southern California, Arizona, Texas, and Florida deal with shorter and warmer winters. Colonies may struggle with unsustainable brood rearing, forage dearth, and spikes of extreme cold weather. The queen's health during this period is crucial, as it sets the stage for the colony's survival and productivity in the subsequent foraging season.

While the queen is generally protected from pathogens by several social immune mechanisms like grooming by her retinue of nurse bees, the secretion of antimicrobial substances in royal jelly^13^, and the isolation within the hive, she is not entirely immune to infections. Viruses that infect honey bees have evolved strategies to also infect queens horizontally through worker contact^14^ and vertically through mating^15^. Thus, most of the pathogens that infect workers can also be found in queens^16–19^, however most infections appear to be innocuous with no overt symptomology^20^. While it’s true that some pathogens have been linked with colony loss^21–24^, the direct effects on queen health are far less understood. Among the viruses that infect queens, Deformed Wing Virus (DWV) is the most widespread and well-known. In the absence of the varroa mite (*Varroa destructor*) DWV normally persists at low levels within a colony with no apparent detrimental effects. It can be found in all life stages and castes, including glandular secretions used to feed developing larvae and members of the hive^14,16,25,26^. When *Varroa* is abundant, there is a dramatic rise in DWV, which is aided in transmission by being directly injected into worker and drone hemolymph during feeding. While *Varroa* do not feed directly on the queen, high mite populations can cause high DWV viral titers in queen tissues. The combination of high viral titers and *Varroa* load is linked to immunosuppression in workers^27^, with the number of workers with overt symptomology (deformed/crippled wings) serving as a predictive marker for colony loss^28^. In extreme cases, newly emerged queens can present with the telltale sign of crippled wings^29^, but subclinical infections on queen health, fecundity, and overall colony dynamics warrants further investigation.

The gut microbiota plays a significant role in nutrient absorption and overall immune function in honey bees. Previous studies have shown that the composition of the queen gut microbiota can change with the age^30,31^, possibly affecting her health and susceptibility to disease. Like workers, the queen microbiota is highly structured and represented by few species which belong to the genera *Bombella*, *Lactobacillus*, *Apilactobacillus*, *Commensalibacter* (Alpha 2.1), *Bombilactobacillus*, and *Bifidobacterium*. Worker-centric genera like *Snodgrassella*, *Gilliamella*, and *Frischella* are sometimes found as either dominance environments (dysbiosis) or relatively low abundance which may indicate they do not serve essential functions in the queen and may even be detrimental. Other honey-bee associated bacteria like *Bartonella* and *Melissococcus*, the causative agent in the brood disease European Foulbrood have not been reported in queens.

Queen health and immunity is intrinsically linked with vitellogenin expression, a molecule that is constitutively expressed throughout the queens’ body, but mostly by the fat body tissue^32,33^. Vitellogenin is an egg-yolk protein, co-opted by honey bees to serve multiple purposes not seen in other animal systems such as immunity, oxidative stress, nutrition, and longevity^33–35^. The fat body in honey bees serves multiple essential functions, akin to the mammalian liver and adipose tissue. It is also a critical site for the production of antimicrobial peptides (AMPs) in response to pathogen associated molecular patterns (PAMPS) of bacterial and fungal origin. Understanding how gene expression in the fat body and other tissues change in response to pathogen pressure is vital for a comprehensive view of queen health. Among the inducible immune-effectors of the honey bee immune response are Peptidoglycan Recognition Proteins (PGRPs), which can recognize and bind to bacterial cell walls, thereby triggering immune responses. A single membrane-bound PGRP in bees (PGRP-LC) acts as a transmembrane receptor upstream of the IMD pathway and is activated by the DAP-type peptidoglycan of Gram-negative bacteria or certain Gram-positive bacteria^36,37^. There are also short PGRPs that recognize lysine-containing peptidoglycan, triggering a proteolytic cascade that activates the Toll pathway. Honey bees also have defenses against viruses through the a Jak/STAT homologue pathway and RNA interference (RNAi)^38,39^. Thus, honey bee immune response is capable of action against bacteria and viruses but can also respond to fungi like *Varimorpha* (*Nosema*) *ceranae*; a microsporidian midgut parasite and the leading cause of dysenteric colony losses worldwide.

The primary aim of this study is to explore variation in queen gut microbiota, host gene expression, and pathogen prevalence associated with overwintering in a warm southern climate within a large-scale commercial beekeeping operation. We seek to understand how factors at the worker and colony level interact, and what implications they may have for queen health. To this end, we expand on a longitudinal study performed in the Imperial Valley of California from September 2016 through January 2017. In previous work it was revealed that colony or worker level factors *Nosema*, *Varroa,* and DWV increased from early to late winter, while the same time period was characterized by decreased Vitellogenin (Vg) and increased AMP expression in workers^40^. Over the same time period, queens showed a sharp increase in mandibular pheromone compliment homovanillic alcohol (HVA) suggesting a shift in queen quality^41^. We suggest that changes in colony level metrics and worker gene expression form the foundation for queen health documented in this study. By collecting interconnected data points on workers, queens, and colonies over time, we seek to produce a comprehensive framework that can better predict changes in colony and queen health.

## Methods

### Experimental design

Queens were obtained from colonies under the management of Ashurst Bee Co. Inc. (Westmoreland, CA), following the methodology previously established by Ricigliano et al., 2018. Briefly, these colonies were distributed across three sites in Southern California: Site 1 (Best 115 E and W, coordinates 33° 01′ 38.8′ ′N 115° 31′ 07.8′′ W), Site 2 (Young and River, coordinates 33° 07′ 58.8′′ N 115° 34′ 05.3′′ W), and Site 3 (Jacobsen 111, coordinates 33° 04′ 30.5′′ N 115° 31′ 14.9′′ W). Each site hosted a balanced mixture of both young and mature Italian queens, all originating from the same commercial breeding source. Young queens were approximately 4 months old and older queens were approximately 16 months old. A carbonyl assay of fat body tissue was also used to confirm biological age. A subset of queens of each age were randomly sampled from each site across the span of September 2016, November 2016, and January 2017 resulting in 96 queen samples over the 5-month period.

### Dissections, protein oxidation assay, and nucleic acid extractions

All 96 queens were collected into sterile 2.0-ml tubes and immediately frozen on dry ice and stored at − 80 °C for nucleic acid extraction. Queens were pinned through the thorax in 70% ethanol to wash and aid in dissection. Micro-dissection scissors were used to cut through the sides of the abdomen to access the digestive tract which was removed and floated in ethanol to separate the gut tissues. The abdominal fat body, along with the attached dorsal sclerites, were extracted as a single unit for use in gene expression and protein oxidation assays, aimed at quantifying biological aging.

The fat body, midgut, and ileum tissues were bead-beaten in 1× TE buffer for 2 min at 30-s intervals and centrifuged to recover the supernatant. The fat body supernatant fraction used in a protein oxidation assay quantifies the accumulation of protein carbonyl groups associated with oxidative stress and aging^42^. This well-defined assay, previously deployed in related studies, was applied in accordance with the methods described in Copeland et al.^43^. To extract nucleic acids (DNA and RNA simultaneously) we used Qiagen AllPrep PowerViral DNA/RNA Kit (Qiagen, Hilden, Germany) following the manufacturer’s protocol and methodology also reported in Copeland et al.^43^.

Using the DNA, we quantified total bacterial abundance for the midgut and ileum tissues using a quantitative PCR (qPCR) assay of the 16 rRNA gene^44^. We created a standard curve using a tenfold serial dilution series of a plasmid standard containing a full-length Escherichia coli 16S rRNA gene. We amplified a 466 bp fragment in the V3–V4 region of the 16S rRNA gene using universal primer pair (5′-CCTACGGGDGGCWGCA-3′ and 5′-GGACTA CHVGGGTMTCTAATC-3′). PCR reactions were performed in triplicate on a BioRad CFX96 (Biorad, Hercules, California, US) as follow: 12 μl reactions containing 9 μl of iTaq Universal SYBR Green Supermix (BioRad, Hercules, California, US), 0.5 μl forward primer,0.5 μl reverse primer, and 2 μl of DNA template. The cycling conditions were 95 °C for 3 min followed by 40 cycles of 95 °C for 10 s and 60 °C for 60 s. The qPCR results were expressed as the total number of 16S rRNA gene copies per DNA extraction (200 μl volume elution). To characterize the microbial communities of the queen midgut and ileum, we amplified the V3–V4 region of the 16S rRNA gene using PCR primers (341F 5′-CCTACGGGNGGCWGCAG-3′; 805R 5′-GACTACHVGGGTATCTAATCC-3′). DNA library preparation was performed following Illumina MiSeq DNA library preparation protocol. Sequencing was performed at the University of Arizona Genetics Core (UAGC) on a MiSeq following the manufacturer’s guidelines. The sequence data for this study have been deposited in GenBank, Sequence Read Archive no. as PRJNA1061232.

### Immune gene expression and quantification of pathogen titers

To quantify mRNA levels, a cDNA template was generated from the purified RNA fraction. Briefly, RNA was converted into cDNA with Thermo Scientific RevertAid First Strand cDNA Synthesis Kit (Thermo Fisher Scientific, Waltham, Massachusetts, United States) following manufacturer’s instructions. PCR reactions were performed using gene-specific primers and the following protocol: initial denaturation at 95 °C for 5 min; 40 cycles with denaturation at 95 °C for 15 s; and a combined annealing and extension step at 58 °C for 30 s. The reactions were carried out using iTaqTM Universal SYBR^®^ Green Supermix (Biorad, Hercules, California, US) in triplicate on an CFX96TM Real-Time PCR Detection System (Biorad, Hercules, California, US).

RNA fractions were used to create cDNA and examine gene expression, *Nosema* intensity, virus abundance, and viral titers qPCR. To confirm the absence of contaminant DNA and primer dimers, we used no-template controls made of water and analyzed melt-curves for each qPCR plate. Relative gene expression was calculated using the 2^–∆∆Ct^ method^45^ using both β-actin and RPS18 as reference genes^46^. Genes were chosen to cover a range of processes associated with innate immunity including; B-Gluc2, DOPA Carboxylate, abaecin, apidaecin, defensin1, defensin2, hopscotch, hymenoptaecin, IRP30, lysozyme1, lysozyme2, lysozyme3, PGRP-LC, PGRP-S1, PGRP-S2, PGRP-S3, PPO, vitellogenin, AmEater, argonaut2, and dicer (Table S1). After normalization, we log-transformed expression levels to approximate normality and tested for differences by month and site using the parametric test, ANOVA with Tukey’s HSD in JMP 14. We also pooled cDNA to quickly screen for honey bee viral pathogens: Acute Bee Paralysis Virus (ABPV), Black Queen Cell Virus (BQCV), Chronic Bee Paralysis Virus (CBPV), Deformed Wing Virus (DWV-A and DWV-B), Israeli Acute Paralysis Virus (IAPV), Kashmir Bee Virus (KBV), Lake Sinai Virus (LSV), Sacbrood Virus (SBV), and Slow Bee Paralysis Virus (SBPV).

Virus titer standards were generated from PCR products that were purified using the GeneJET PCR Purification Kit (Thermo Scientific, Waltham, MA), ligated using pGEM^®^-T Easy Vector Systems (Promega, Madison, WI) to yield plasmid vectors, which were then transformed into DH5α™ Competent Cells. Transformed colonies were selected and grown overnight in broth. Plasmids were isolated using the GeneJET Plasmid Miniprep Kit (Thermo Scientific, Waltham, MA) and quantified using an Implen nanophotometer P300. The known mass of the plasmid plus PCR insert was used to calculate DWV plasmid-standard copies per µL. Primers for immune genes and pathogens can be found in Table S1^26,47–50^.

### 16S rRNA gene community analysis

16S rRNA gene sequence data were processed using MOTHUR v.1.44.343^51^ according to previously published protocols^43^. Briefly, paired end reads were joined using the make.contigs command. We used the SED command in UNIX to remove the first and last five nucleotides from each sequence. Sequences were then screened to remove ambiguous bases, using the screen.seqs command. Unique sequences were generated using the unique.seqs command. A count file containing group information was generated using the count.seqs command. Sequences were aligned to the BEExact database^52^ using the align.seqs command. Sequences were filtered to remove overhangs at both ends and gaps using filter.seqs. The unique.seqs command was ran again to remove new redundancies from filtering. A precluster step using pre.cluster was performed followed by the chimera.uchime command^53^ to remove chimeric sequences. Sequences not of bacterial origin (fungi, archaea, mitochondria, and chloroplasts) were removed using the remove.seqs command. All unique sequences with one or two members (single/doubletons) were removed using the AWK command in UNIX. A distance matrix was constructed for the aligned sequences using the dist.seqs command. Sequences were classified at the unique level with the BEExact database using classify.seqs command. Unique sequences were then merged at the species-level with the merge.otus command.

We curated the data to remove sources of contamination associated with low abundance DNA environments^54^. Queen libraries, especially the midgut may contain very little DNA and contamination skews results. Following criteria previously established^55^, we used three exclusion criteria to identify contaminant OTUs: (1) strong negative Pearson’s correlations with read number and microbiome size based on 16S rRNA sequences (BactQuant), (2) intercorrelations with other known or suspected contaminants, and (3) association with contamination from other works^54^. Additionally, we corroborated our contamination with kit controls and the R program *decontam*^56^.

### Statistical analyses

The 13 most abundant operational taxonomic units (OTUs) and a sum of remaining OTUs were normalized by qPCR BactQuant absolute abundances by first calculating the proportion of each OTU by dividing the raw read count into the total number of sequences per sample. Each ratio was multiplied by the total BactQuant 16S rRNA gene copies qPCR for each sample. Next, each OTU was corrected for 16S gene copies per bacterial cell; 16S rRNA gene copy number were assigned based on the exact match or closest taxonomic representative^57^. OTUs without a close taxonomic representative in the database was assigned 4.2 gene copies, the mean 16S rRNA gene copy number^58^. Next, the data were CLR-transformed using the software CoDaPack^59^.

A two-way multivariate analysis of variance (MANOVA) was performed on CLR-transformed data with OTUs 1–15 as dependent variables. The MANOVA examined sampling month and site as independent variables, and post hoc pairwise analyses were conducted using Tukey honestly significant difference (HSD) test. Normalized absolute abundance data, gene expression, and viral titers were also analyzed using ANOVA with Tukey HSD by both sampling month and site. When appropriate, a false discovery rate (FDR) was employed to account for multiple comparisons. We examined carbonyl as a continuous variable that serves as a proxy for biological age. These values were used in a bivariate analysis against bacterial cell numbers and gene expression. These were analyzed with ANOVA and corrected with FDR for multiple comparisons.

We also used Pearson’s correlations as a statistical measure to quantify the strength and direction of a linear relationship between two continuous variables. In our case, we explore patterns between OTU abundance, gene expression, viral titers, and microbiome size. The coefficient ranges from − 1 to 1, with values closer to 1 indicating a strong positive linear relationship, values closer to − 1 indicating a strong negative linear relationship, and values around 0 suggesting little to no linear correlation. To further explore the distribution of these correlations, we categorized them into different levels of significance based on the value of the Pearson's coefficient *R*. For positive correlations, 'Strong Positive' was defined as *R* ≥ 0.8, 'Moderate Positive' as 0.8 > *R* ≥ 0.5, and 'Mild Positive' as 0.5 > *R* > 0. Similarly, for negative correlations, 'Strong Negative' was defined as *R* ≤  − 0.8, 'Moderate Negative' as − 0.8 < *R* ≤  − 0.5, and 'Mild Negative' as − 0.5 < *R* < 0. Correlations close to zero and not significant were categorized as 'Not Significant'. We visualized these data as stacked bar charts, tallying the number of correlations falling into each category for different tissues (midgut, ileum, and fat body) and across different months (September, November, and January). To statistically assess the variations in these distributions across months, we performed Chi-Square Tests for Independence. This allowed us to determine whether the distribution of significance levels was consistent across the different months for each tissue type. We calculated the expected frequencies for each cell in the contingency table and used these to compute the Chi-Square statistic and associated *p*-value. Cells contributing significantly to the Chi-Square statistic were further identified using standardized residuals, calculated as $\frac{(Observed-Expected)}{\sqrt{Expected}}$. Standardized residuals greater than 1.96 or less than − 1.96 were considered to significantly contribute to the Chi-Square value, indicating that the observed frequency was significantly different from the expected frequency under the assumption of independence between months and significance levels.

## Results

### Sequence read curation and microbial community analysis

Next generation sequencing provided 9,532,084 raw reads (400 bp) across 192 libraries; half were queen midguts and the other queen ileums (Table S2). The BEExact classifier matched many sequences to the species-level^52^, however, most *Bombella* sequences were classified as *Bombella_unclassified*, while *Bo_apis* was sparse with only 33% of samples having any reads even though it was the 7th most abundant OTU. For downstream analyses we combined these into *Bombella sp.* After quality filtering and removal of laboratory contamination (Table S3), 8,002,199 reads were conserved accounting for 98.23% of sequences and 14 operational taxonomic units (OTUs) (Table S4): *Bombella *sp., *Lactobacillus paniaspium*, *Commensalibacter *sp*.* (Alpha 2.1), *Lactobacillus apis*, *Snodgrassella alvi*, *Lactobacillus helsingborgensis*, *Frischella perrara*, *Apilactobacillus kunkeei*, *Bombilactobacillus mellis*, *Lactobacillus kimbladii*, *Lactobacillus *sp*.*, *Bifidobacterium asteroides*, *Bombilactobacillus mellifer*, and *Lactobacillus melliventris*. The remaining 144,429 reads and 1.77% of sequences were conserved and summed as the 15th OTU. The average read depth was 43,359 per library, (29,296 for midguts and 57,277 for ileums). The average size of the midgut based on 16S rRNA gene copies was 5.33E+04. Queen ileum tissues averaged 1.15E+06 16S rRNA gene copies. The size of the queen microbiota was driven largely by the top 4 OTU, *Bombella *sp., *L. panisapium*, Alpha 2.1, and *L. apis* which accounted for 84% of total sequences in the curated dataset.

### Queen microbiota

**Figure 1:**
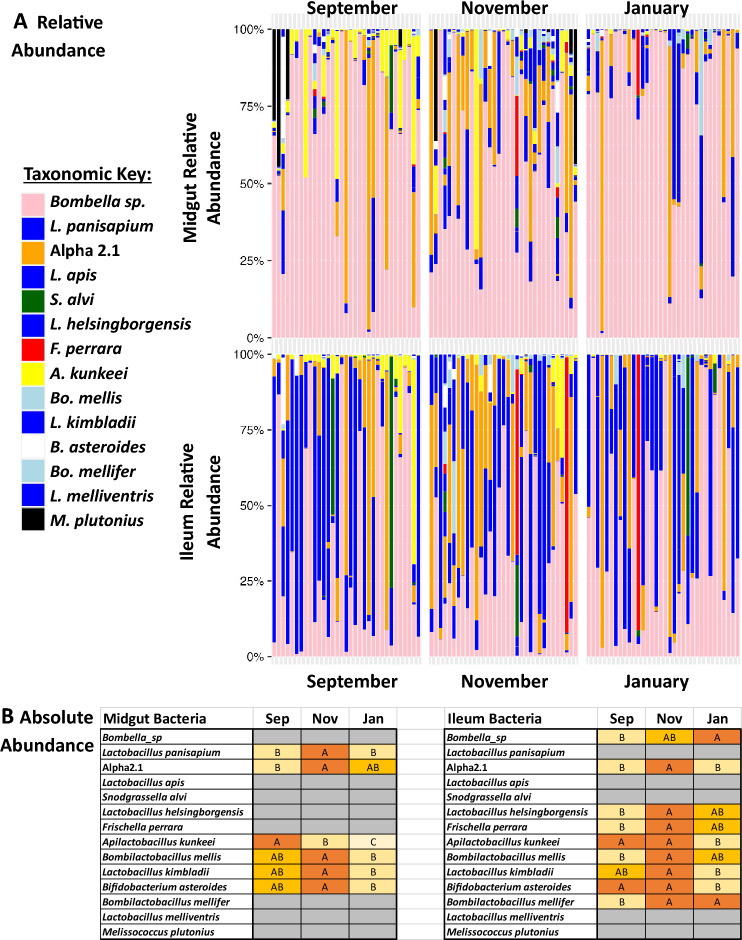
Relative and absolute abundance of midgut and ileum microbiotas. (**A**) The relative abundance of the honey bee queen midgut and ileum microbiotas by month. Color-coded bars represent relative abundance that has been corrected by species-specific 16S rRNA gene. (**B**) Absolute abundance statistical test with ANOVA Tukey HSD, ordered letters report. A is always highest abundance/expression. Levels not connected by same letter are significantly different. Gray = did not significantly differ.

Queen midgut tissues were dominated by *Bombella *sp. (Fig. 1) but differed significantly by month in the MANOVA model (Table S5). The MANOVA uses CLR-adjusted values to interpret significant differences in the relative abundance of bacteria strains. *L. helsingborgensis*, *A. kunkeei*, and *B. mellifer* were significant under the MANOVA model. Microbiota size, *Bombella *sp., *L. apis*, and *B. asteroides* all increased with biological age measured through fat body carbonyl contents (Table S6). Absolute abundance generally saw patterns of highest abundance in November for 6 of the 15 OTUs (Fig. 1)*. A. kunkeei* was the only bacteria that decreased over the sampling period, a pattern also observed in the relative abundance MANOVA (Fig. 1, Table S5). Notably, five queen midguts harbored a relative abundance of *Melissococcus plutonius* at greater than 25%.

In queen ileums we found a significant effect by month and site, but not as an interaction effect in the MANOVA model (Table S5). For month, the dependent variables that were significant were *Bombella *sp., *A. kunkeei*, and *B. asteroides* and despite site being significant in the overall model, after FDR correction no dependent variables met the significance threshold of p < 0.05. Microbiota size, *Bombella *sp., *L. panisapium*, and *B. asteroides* increased with biological age measured through fat body carbonyl contents (Table S6). The absolute abundance differences in the ileum generally matched the midgut, with most OTUs having the highest abundance in November, however 10 out of 15 OTUs differed significantly (Fig. 1). Interestingly, we also found that *Bombella *sp*.* absolute abundance increased through the sampling period with the highest levels in January.

### Gene expression

**Figure 2:**
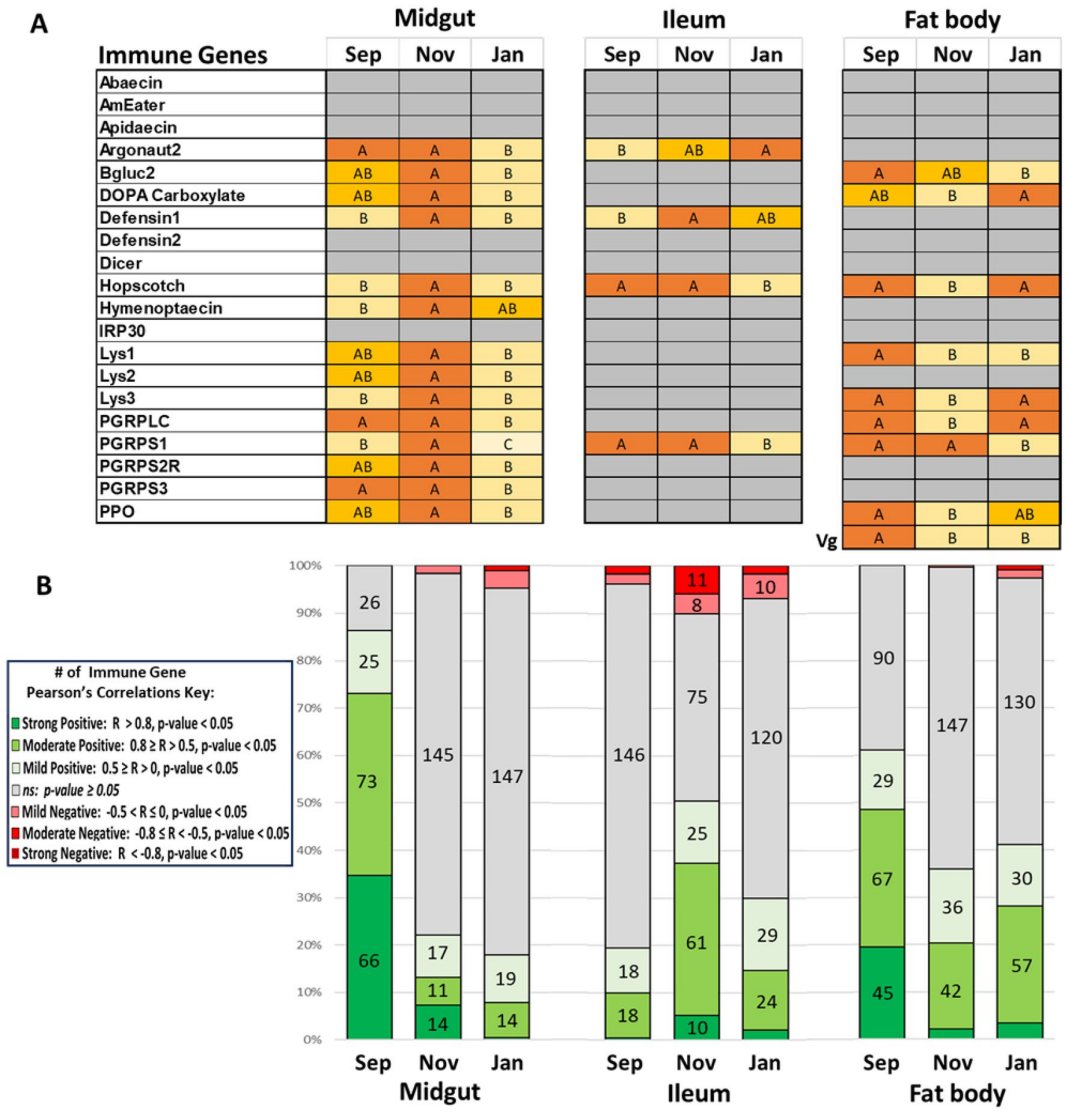
Gene expression and correlations. (**A**) Gene expression reported by month using ANOVA Tukey HSD, ordered letters report. A is always highest abundance/expression. Levels not connected by same letter are significantly different. Gray = did not significantly differ. (**B**) Stacked bar chart showing the number of statistically significant Pearson’s correlations by tissue and month.

We quantified gene expression in the midgut, ileum, and fat body (Fig. 2, Figure S1). We found differential gene expression in all tissues, but the largest differences were in the midgut and fat body. Starting with the midgut, nearly all genes peaked in expression levels during the month of November. This included genes *argonaut2* and *hopscotch* which are involved in RNA interference and immune signaling, respectively. Antimicrobial peptides *defensin1*, *hymenoptaecin*, and *prophenol oxidase* (*PPO*) which is involved in the melanization pathway. Several lysozymes and peptidoglycan recognition proteins (PGRPs) were also upregulated in November, which are typically innate immune responses to the peptidoglycan layer found in bacterial cell walls. Pearson’s correlations of gene expression in the midgut were extremely positively intercorrelated (Fig. 2; Table S7). During November when gene expression levels are highest, the number of significant positive intercorrelations are severely suppressed in November and January. We used a Chi-Square Tests for Independence to determine if there is a significant association between two categorical variables: the month (MG Sep, MG Nov, MG Jan) and the number of significant Pearson's correlations (Strong Positive, Moderate Positive, etc.). The midgut had a high Chi-Square statistic (817.31, *df* = 12, *p *value < 0.0001) reinforcing the result of a strong departure from independence between the two variables (Table S8) with the number of positive correlations contributing most to the statistic. The remaining strong positive Pearson’s correlations (*R* > 0.8) in November are related to the genes *Beta-glucanase2* and *DOPA carboxylase*. Not only are these genes highly intercorrelated (*R* = 0.8039, *p value* < 0.0001), they also both form strong positive correlations with *Lysozyme1*, *Lysozyme3*, *PGRPS1*, and *PPO*. By January there is only one strong correlation remaining: *PRPLC* and *Lysozyme1* (*R* = 0.847 *p value* < 0.0001). The number of negative correlations also increased throughout the sampling period, for example by January there’s a moderate correlation between *DOPA carboxylase* and *hymenoptaecin* expression (*R* = − 0.6034, *p value* = 0.0002).

Ileum gene expression was largely static between months outside of *argonaut2*, *defensin1*, *hopscotch*, and *PGRPS1* which showed variable differential patterns (Fig. 2, Figure S1). *Argonaut2* expression increased over the sampling months, with highest levels in January. *Defensin1* peaked in November, which was like the expression pattern for the midgut. *Hopscotch* and *PGRPS1* expression were static in September and November but dropped significantly in January. Despite low differential expression, the highest number of significant correlations was found in November (Fig. 2), while both September and January had low correlations matching the later months of the midgut (Table S7). The ileum also showed a substantial Chi-Square statistic (435.71, *df* = *12*, *p value* < 0.0001), with the number of non-significant correlations between the months contributing the most to the statistic (Table S8). Ileums in November also had the most moderate negative correlations in the dataset, with some nearly meeting our threshold for strong negative correlation. These moderate negative correlations (*R* <  − 0.7, *p value* < 0.0001) are with *PGRPS3* and several viral signaling and immune genes: *argonaut2, dicer*, *hopscotch*, and *Lysozyme3*. *PGRPS2R* also had moderate negative correlations (*R* <  − 0.5, *p value* < 0.005) with the same genes. Correlations in September and January are much sparser. In September, all of the strong positive correlations disappear, and *dicer* has moderate negative correlations (*R* <  − 0.5, *p value* < 0.0011) with *DOPA carboxylase*, *PGRPS1* and *PGRPS2R*.

In contrast to the midgut and ileum, the fat body showed more variable gene expression patterns, with the overall pattern showing decreased expression in November relative to September and January for six out of eight significant genes (Fig. 2, Figure S1). The six genes which were suppressed in September were *DOPA carboxylase*, an enzyme that catalyzes the decarboxylation of DOPA (3,4-dihydroxyphenylalanine) to dopamine, a neurotransmitter, *hopscotch*, *lysozyme3*, *PGRPLC*, and *PPO*. *Beta-glucanase2*, an enzyme that breaks down beta-glucans, complex sugars found in the cell walls of fungi, bacteria, and some plants decreased over the sampled season. Similar to the midgut, September had the most significant correlations which decreases over the sampled time period (Fig. 2, Table S9). The fat body also had a substantial Chi-Square statistic (508.85, *df* = *12*, *p value* < 0.0001), with the changes in the number of positive correlations between the months contributing most to the statistic. Nearly all strong positive correlations disappear from September to November, except *hymenoptaecin* with *PGRPS2R* (*R* = 0.8383, *p value* < 0.0001) and *PGRPS3* (*R* = 0.7734, *p value* < 0.0001) which seemingly persist through January. January also added strong positive correlations: *argonaut2* with *hymenoptaecin* (*R* = 0.7831, *p value* < 0.0001) and *PGRPS2* (*R* = 0.8571, *p value* < 0.0001).

### DWV, *Nosema*, and European Foulbrood

**Figure 3:**
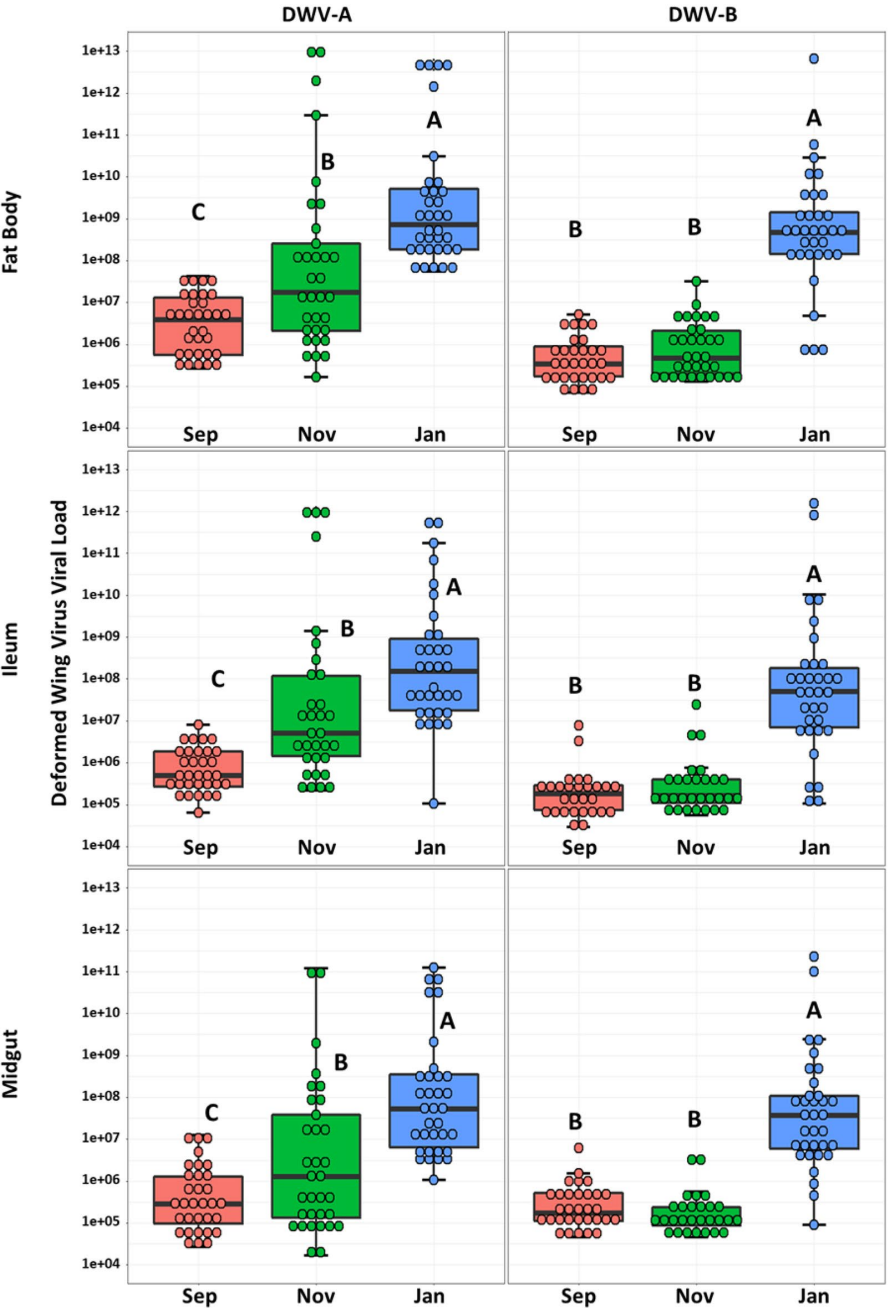
DWV. Comparison between expressions of DWV-A and B by tissue type and month. Y-axis is log-scale total viral copies. Different letters indicate significant differences (p < 0.05).

Our initial screening for viruses yielded mostly negative results for Acute Bee Paralysis Virus (ABPV), Chronic Bee Paralysis Virus (CBPV), Israeli Acute Paralysis Virus (IAPV), Kashmir Bee Virus (KBV), Lake Sinai Virus (LSV), Sacbrood Virus (SBV), and Slow Bee Paralysis Virus (SBPV). We found variable levels of Black Queen Cell Virus (BQCV) just above our limit of detection, so we excluded this and all negative viruses from downstream analysis due to low prevalence and abundance. Deformed Wing Virus (DWV-A and DWV-B) was found in all tissues tested: midgut, ileum, and fat body (Fig. 3). Over the course of the sampling period, DWV-A increased by several orders of magnitude in all tissues. DWV-B matched DWV-A levels for September for both September and November, but in January increased by several orders of magnitude in all tissues. Individual viral titers were similar across all tissues, for example high titer samples were high across all three tissues. Carbonyl accumulation was not a factor in DWV titers.

We also checked Pearson’s correlations of DWV-A and -B with gene expression and total brood area. When DWV-A levels begin to rise in November, the midgut had the strongest immune response across all tested gene expression (Fig. 2, Table S11). *Hymenoptaecin* had a moderate positive correlation (*R* = 0.6692, *p value* < 0.0001) that becomes more potent in January (*R* = 0.7604, *p value* < 0.0001) along with RNAi-pathway gene *argonaut2* (*R* = 0.7041, *p value* < 0.0001). The pattern with these genes exists in the fat body as well, but includes moderate positive correlations (*R* > 0.5, *p value* < 0.024) for the antimicrobial peptides *apidaecin* and *defensin1* and PGRPs *PGRPS2R* and *PGRPS3*. Correlations with DWV-B during the spike in January are sparse with most only being mild correlations, (*R* < 0.5). There were mild negative correlations (*R* > 0.5, *p value* < 0.035) of DWV-A and -B with total brood when sampled and the mean total brood across the sampling period (Table S12).

**Figure 4:**
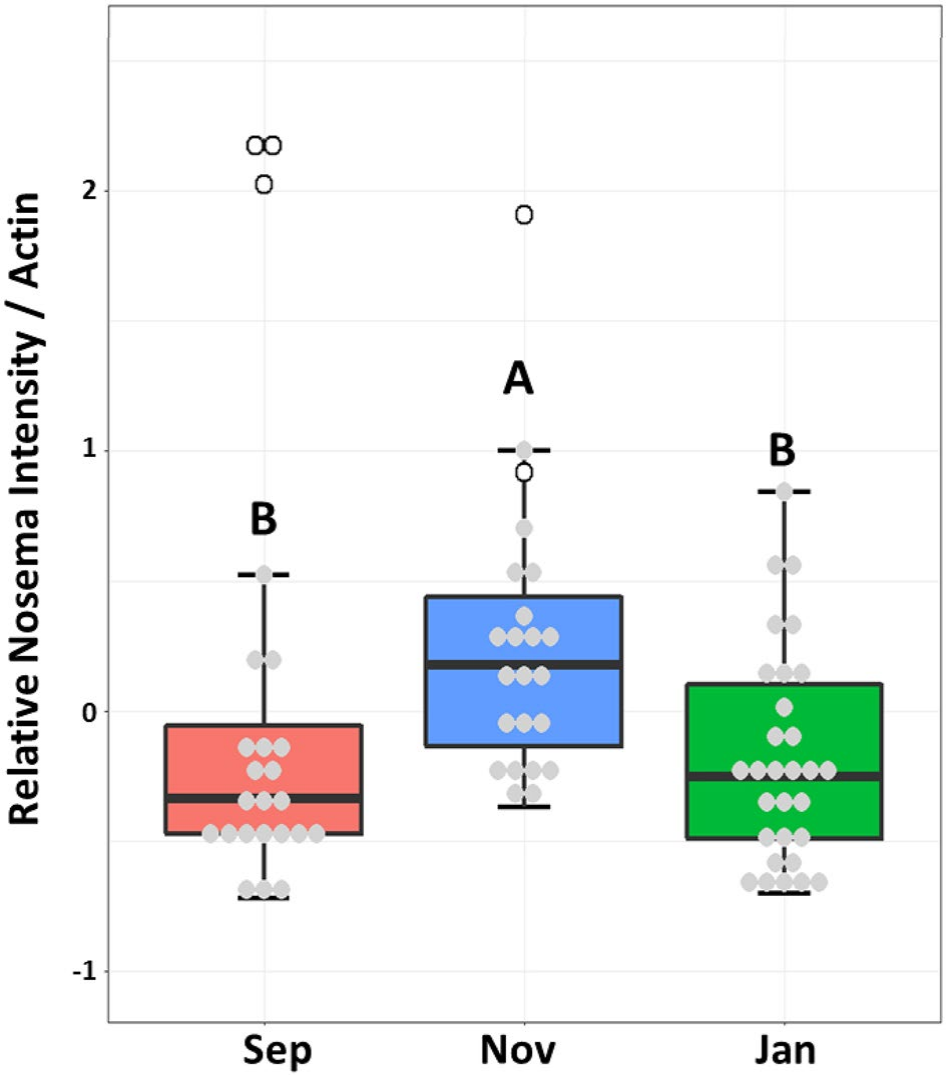
Nosema and *Melissococcus* relationship. Relative *Nosema* intensity in queen midguts. The open circles are queens from Site 1 with high levels of *Melissococcus plutonius*. Different letters indicate significant differences (p < 0.05).

*Nosema* intensity was measured in the midgut across the season (Fig. 4). Overall *Nosema* intensity was low and not different between the months, however there were five out of six samples with the highest *Nosema* burden that all came from Site 1 and were sampled in September and November. We examined gene expression and did not find any significant correlations between *Nosema* intensity and the expression levels of the immune-related genes we analyzed in these five samples. The microbiota of these five queens also had high (< 25%) relative abundance of *Melissococcus plutonius*, the causative agent in European Foulbrood. The sixth queen with higher *Nosema* burden also came from Site 1 in November but did not carry *M. plutonius* greater than 1% relative abundance.

## Discussion

Queen health is a critical measure of a successful colony, especially during stressful events such as disease, dearth, or overwinter. However, it is too costly to sacrifice the queen, such that her health is typically inferred from the way workers treat the queen, egg laying capability, brood patterns, and other colony level metrics. We designed this experiment to reveal changes in the gut microbiota, host gene expression, and pathogen pressure associated with overwintering in a warm southern climate in a large-scale commercial beekeeping operation. Our results support the hypothesis that overwintering has a systemic effect, allowing queen health to be predicted based on worker characteristics, pathogen levels, and colony metrics.

Although queen age was not a factor in this study, we used oxidative damage accumulated in the fat body tissue as a proxy for chronological age. Each sampling month had an equal number of young and old queens, and overall differences in microbiota were minimal. When we regressed carbonyl accumulation against our top OTUs we found that biologically older queens had larger microbiotas and more *Bombella *sp. and *Bifidobacterium* in both the midgut and ileum tissues. *Lactobacillus apis* also increased with age in the midgut, while *Lactobacillus panisapium* increased with age in the ileum highlighting strain specificity and diversity within the tissues. The increase in abundance of *Lactobacillus *spp. and *Bifidobacterium* align with previously reported studies on queen microbiota aging^30,31,60^. Generally, the relative abundance of species rarely differed significantly between months. *Apilactobacillus kunkeei* is one standout from midguts which decreased over the sampling period. *A. kunkeei* is a fructophilic lactic acid bacteria that prefers fructose as its primary carbon source^61^. Its decrease over the sampling period could be indicative of changes in the dietary intake of the queens, a shift in the microbial community dynamics within the midgut, or a change in the queens metabolism as a result of a low sugar diet.

The absolute abundance of many OTU peaked in the ileum during November, and these tissues were dominated by top OTUs *Bombella*, *L. panisapium*, Alpha 2.1, and *L. apis*. The ileum appeared more stable in terms of relative abundance, but there were some dominance libraries of *Snodgrassella alvi* and *Frischella perrara* which are broadly considered worker bacteria. In workers, *S. alvi* interfaces with the ileum epithelial tissue, consumes available oxygen, and forms a biofilm with *Gilliamella* and *L. apis*^62,63^. *S. alvi* appears to be tolerated by queens to some degree, apparently replacing Alpha 2.1^30,31^. *F. perrara* causes a scab formation in the worker pylorus^64^, but we did not detect any tissue abnormalities during dissection.

Queens are protected by several social immune mechanisms, such as the retinue of workers that groom and feed her with the diet of royal jelly. Despite the highly antimicrobial diet and the protection from mostly living in the hive, she is still vulnerable to pathogens through horizontal transmission^17^ and vertical transmission during mating nuptial flights^14,15^. Multiple virus infections in queens are common^16^, yet it’s unclear how these infections effect health, behavior, and overall physiology of queens as overt symptomology often remains elusive. Out of three tissues tested, the fat body had the highest DWV viral titers in January which correspond to hive *Varroa* levels and nurse DWV intensity from the same hives and workers associated with these queens^40^. DWV titers correlated between tissues, such that queens with high titers in the fat body were also high in the ileum and midgut suggesting systemic infection. In honey bees, the fat body provides many of features of the mammalian liver and adipose tissue, fulfilling essential metabolic functions such as the production of antimicrobial peptides and vitellogenin^33^. Vitellogenin is synthesized in the fat body and transported to the ovaries, where it is incorporated into developing oocytes^65^. Beyond its role in reproduction, vitellogenin also has immunomodulatory functions and is involved in lifespan regulation and stress resistance in honey bees^35,66^. Given that DWV replication occurs in the cytoplasm and plasma membrane of fat body cells^67^, this could impair the queen’s immune function and fecundity, potentially leading to the uptick in yearly queen failures experienced by commercial beekeepers. One large study surveyed honey bee queens and found coinfections of both DWV-A and -B, but determined they had little impact on the health and functional status of the queen despite instances of degenerated ovaries^18^. However, even with the overall patterns of depressed gene expression in the fat body during November, queens with the highest viral loads expressed greater antimicrobial peptides and PGRPs, and the lowest expression of vitellogenin hinting that covert DWV infections may impact queen health. Workers overwintering experience a reduced expression of immune genes and higher DWV loads^68^, suggesting queens could be responding in a similar manner. DWV-B is considered the more virulent strain^69^ replacing A across the US^70^, but in our data DWV-B lagged behind DWV-A.

In a separate queen cohort study sampled during the same overwintering time points from the same beekeeping operation, Carroll et al. found that queen fat body protein contents were lowest in September and November, while lipid contents were highest in November and January^41^. These findings suggest a complex interplay between viral infections, metabolic status, and immune response. The elevated expression of antimicrobial peptides and PGRPs in queens with high viral loads may represent a compensatory immune response to the viral infection. However, the concomitant low expression of vitellogenin could indicate a trade-off between immune function and reproductive capabilities. This is supported by the negative correlations between brood area and DWV viral titers. The data from Carroll et al. further complicates the picture, showing that protein and lipid reserves in the queen fat body fluctuate during the overwintering period, potentially affecting both immune responses and reproductive output. These metabolic changes could be influenced by or be a response to the viral infections and may serve as an additional stressor that impacts queen health.

The relationship between DWV titers and gene expression of the midgut from September to January was also complex and dynamic. DWV did not reach the same levels of infection as it did in the fat body, but replication in the guts of queens is documented^67^. In September, when DWV titers are lowest, there were 164 significant positive correlations between different genes, suggesting a coordinated and possibly effective immunity prior to rampant *Varroa* pressure and high levels of DWV infection over the season. As DWV titers increase in November and peak in January, the number of significant correlations among immune genes drops dramatically to 42 and 34, respectively. This indicates a disruption in the immune system's coordinated response with several potential explanations. The immune system might be reallocating resources as the viral infection progresses, leading to a more complex or less straightforward relationship between the expression of different immune genes. In response to co-infections, the immune system allocates resources to fight multiple pathogens simultaneously. In such cases, the immune system often prioritizes one pathogen over another, leading to a more complex or less straightforward relationship between the expression of different immune genes. This has been observed in co-infections with parasites and bacteria^71^, or between different viral infections^72^. It's also possible that the virus has evolved mechanisms to disrupt the host's immune response^73^, especially as the viral load increases^25^. Lastly, there may be a threshold level of DWV that, once exceeded, disrupts the normal, coordinated immune response in queens, however this has not yet been established. Unfortunately, our design did not distinguish between these alternative hypotheses.

Lysozymes and peptidoglycan recognition proteins (PGRPs) are mainly geared towards bacterial defense and are not directly involved in combating viral infections like DWV. However, there are several indirect ways these molecules could be relevant in the context of a DWV infection. For instance, a DWV infection could weaken the bee's immune system, making it more susceptible to bacterial infections. Indeed, the midgut gene expression seems to indicate an active response to a perceived pathogen during November, which suggests bacterial opportunism. In *Drosophila melanogaster*, immune response to pathogens is highly regionalized in the gut with the Toll pathway active in the foregut and hindgut, and IMD regulating the midgut^37^. This may explain the robust immune response seen in queen midguts through the Toll and IMD pathways, as most gene expression of the ileum was not significantly different. Recent research suggests that Toll and IMD pathways may also contribute to antiviral defenses. For example, some studies on *Drosophila* have suggested these pathways can be activated by specific viral infections and may help curb viral replication^74,75^. The increased expression of both PGRP-LC and short PGRPs partly explain the high activation of immune pathways. The midgut, as the primary site of food digestion and absorption, is frequently exposed to potential pathogens, so it's not uncommon for it to have a robust immune response. In immune trade-offs, a robust response requires substantial energy, whereas the queen may allocate resources to the midgut at the expense of other tissues, including the fat body. In *Drosophila*, the midgut acts as a sentinel tissue, detecting pathogens first and conveying the signal to the rest of the body^76^ which may explain the delay in fat body gene expression. There’s also a possibility of an active infection of worker nestmates affecting honey bee queen immunity through exposure to cuticular hydrocarbons (CHCs). Immune-challenged workers present immunological cues through CHCs that increase queen expression of AMPs^77^. *Varroa* mites and DWV infection also influence CHC expression^78,79^ and high viral titers and *Varroa* load present with immunosuppressed workers^27^. It’s also possible that if the infection is localized primarily in the midgut, other tissues like the fat body might not be stimulated to the same degree. DWV can actively suppress host immunity by modulating the host immune response, downregulating the expression of immune-related genes in certain tissues to aid their own survival^73^. It’s also possible that different tissues respond to infections in different ways, such that the fat body is mounting a different type of immune response that does not involve the upregulation of the particular genes we explored. In such scenarios, lysozymes and PGRPs might be upregulated as part of a broader immune response. Additionally, while not antiviral themselves, the expression of these molecules could be influenced by the overall activation of the immune system in response to the viral infection or opportunism. A DWV infection could also disrupt the gut microbiome, which might trigger a response from bacterial defense mechanisms like lysozymes and PGRPs. We found significant differences in the absolute abundance of several core microbial members in November midguts, however, studies on queen host-microbe interactions are limited and these results should be interpreted cautiously.

Colonies heavily infected by *Nosema ceranae* often experience excessive levels of queen supersedures^17,49,80^. Queens are partially resistant to *N. ceranae*^17^, and all of our queens had relatively low *Nosema* infection rates, even though relative worker *Nosema* loads rose precipitously in January^40^. However, our queens with the highest *Nosema* loads also revealed heavy loads of *M. plutonius* suggesting either a co-infection or secondary infection had taken place. Our findings indicate a potential link between *Nosema* and *M. plutonius* infections in queens, raising the possibility of synergistic or facilitative interactions between these pathogens that could exacerbate their individual effects. This is supported by the fact that both pathogens target the midgut, a key site for nutrient absorption and immune function. During a *N. ceranae* infection, queens present similar lesions in the epithelial cells of the midgut as workers and with high enough spore counts can succumb to death^17^. *M. plutonius* causes the brood disease European Foulbrood (EFB)^81^. EFB infects unsealed brood when freshly hatched larva ingest food contaminated with *M. plutonius. M. plutonius* proliferates rapidly in the midgut causing it to twist, shrivel, and decompose into a brown/black scale. Prevalence of *M. plutonius* can be high in asymptomatic colonies—one study using qPCR found 90% of colonies within an apiary containing EFB symptomatic apiaries had adult bees that carried *M. plutonius*^82^. Even though adult workers can carry *M. plutonius*, they do not show symptoms of disease, and queens may be similarly unaffected. However, this is the first study to find *M. plutonius* at such high relative abundances in queens to our knowledge. In general, the coincidence of *Nosema* and *M. plutonius* in queen guts may reflect the environmental similarities of queen and larval guts, as both appear to support aerobes in the presence of pure royal jelly, a substance packed with broad-spectrum antimicrobial activity^13^. Members of the microbiome shared by queen and larval guts may also facilitate the transmission of pathogens like *Nosema* and *M. plutonius* between distinct host environments within the colony.

## Conclusion

Our work highlighted the complex interplay between the microbiota, Deformed Wing Virus (DWV), gene expression, and immune responses. We suggest that high DWV loads could disrupt the queen’s coordinated immune response, potentially leading to immune exhaustion or resource reallocation. Its currently unknown whether queens can overcome DWV infections. It should also be noted that if there are high levels of colony viral infection, likewise the queen probably suffers too. These findings are particularly relevant given the high prevalence of multiple viral infections in queens and their potential impact on queen health and hive stability. Furthermore, our results indicate a potential link between *Nosema ceranae* and *Melissococcus plutonius* infections in queens, suggesting that pathogen opportunism is not confined to just workers. These relationships are complex and are dependent on many additional factors like season, location, hive social immunity, and individual queen fitness. Future research should aim to extend these findings through longer-term studies to determine if DWV viral titers recede and how other pathogens like *Nosema* and *M. plutonius* can parasitize queens. Overall, the multifaceted interactions between the microbiota, pathogens, immune responses, and metabolic status presented here highlight the utility of a holistic colony-level approach to understanding queen health and resilience.

### Supplementary Information

Supplementary Information.

## Data Availability

The dataset generated for this study can be found in the GenBank, Sequence Read Archive as PRJNA1061232.
